# Seed germination ecology of hood canarygrass (*Phalaris paradoxa* L.) and herbicide options for its control

**DOI:** 10.1038/s41598-022-19418-8

**Published:** 2022-09-09

**Authors:** Vicent Kibasa, Gulshan Mahajan, Bhagirath Singh Chauhan

**Affiliations:** 1 School of Agriculture and Food Sciences (SAFS), The University of Queensland, Gatton, QLD 4343 Australia; 2 The Centre for Crop Science, Queensland Alliance for Agriculture and Food Innovation (QAAFI), The University of Queensland, Gatton, QLD 4343 Australia; 3 Punjab Agricultural University, Ludhiana, Punjab 141004 India; 4 The Centre for Crop Science, Queensland Alliance for Agriculture and Food Innovation (QAAFI), School of Agriculture and Food Sciences (SAFS), The University of Queensland, Gatton, QLD 4343 Australia; 5 Department of Agronomy, Chaudhary Charan Singh Haryana Agricultural University, Hisar, Haryana 125004 India; 6 Present Address: Namtumbo District Council, Namtumbo, Ruvuma, Tanzania

**Keywords:** Ecology, Plant sciences

## Abstract

Hood canarygrass (*Phalaris paradoxa* L.) is a problematic weed in winter crops of Australia. Experiments were conducted to determine the effects of environmental factors on seed germination of *P. paradoxa* and wheat (*Triticum aestivum* L.) and herbicide options for *P. paradoxa* control. Results revealed that *P. paradoxa* had higher germination (> 89%) at a temperature range from 15/5 ℃ to 25/15 ℃ [day/night (12 h/12 h] compared with 30/20 ℃ and 35/25 ℃. At a temperature regime of 30/20 ℃, *P. paradoxa* had 1% germination; however, wheat at this temperature range resulted in 79% germination. Exposure of seeds of *P. paradoxa* to > 150 ℃ pretreatment (radiant heat for 5 min) resulted in no germination. These results suggest that infestation of *P. paradoxa* could be reduced by residue burning or by planting wheat crops early in the season when the temperature is relatively high. At a water potential of -0.8 MPa, seed germination of *P. paradoxa* and wheat was 75 and 96%, respectively. Similarly, at the highest salt concentration (200 mM sodium chloride), seed germination of *P. paradoxa* and wheat was 73 and 79%, respectively. These observations suggest that like wheat, *P. paradoxa* is also highly tolerant to water and salt stress conditions, therefore, it could invade the agro-ecosystem under water and salt stress situations. Germination of *P. paradoxa* was found to be low (10%) on the soil surface, suggesting that no-till systems could inhibit the germination of *P. paradoxa*. Pre-emergent (PRE) herbicides, namely cinmethylin, pyroxasulfone, and trifluralin, provided 100% control of *P. paradoxa*; however, in the presence of 2 t ha^−1^ of residue cover, pyroxasulfone provided better control of *P. paradoxa* compared with other herbicides. Post-emergent (POST) herbicides clethodim, haloxyfop, and paraquat provided excellent control of *P. paradoxa*, even if the plant size was large (10-leaf stage). Knowledge generated from this study will help in strengthening the integrated management of *P. paradoxa*.

## Introduction

Hood canarygrass (*Phalaris paradoxa* L.) is a major problematic weed in winter season crops of Australia^1^. It grows very well in alluvial, sandy-clay, or clay texture soils with high soil moisture conditions^2^. It has been observed that the high infestation of *P. paradoxa* in wheat (*Triticum aestivum* L.) paddocks of Australia could cause a 40% reduction in yield^3^. These observations suggest that *P. paradoxa* is a very competitive weed in wheat crops. In South Asia, it was observed that heavy infestation of *P. minor* (close relative species of *P. paradoxa*) could cause an 80% reduction in wheat yield^4^. It has been estimated that one plant of *P. paradoxa* can produce up to 21,000 seeds per plant in fallow conditions^5^ (www.crop.bayer.com.au). High seed production and seed shattering tendency of *P. paradoxa* could lead to reinfestation of this weed in paddocks. Under field conditions, *P. paradoxa* produced 3100 seeds m^-2^ in wheat and 50 seeds m^-2^ in barley (*Hordeum vulgare* L.)^6^.

*Phalaris paradoxa* is very similar to *P. min*or in appearance; however, *P. minor* has one sterile floret per spikelet whereas, *P. paradoxa* has two sterile florets per spikelet^7^. Both *P. minor* and *P. paradoxa* originated from the Mediterranean^1^. *Phalaris paradoxa* has become a serious weed in Australia due to distinct attributes such as its high seed production, innate dormancy, periodicity in emergence patterns, and the adoption of no-till agriculture^6^. As a result, this weed becomes the third most difficult grass weed after *Avena fatua* L. and *Avena ludoviciana* (wild oats) in the northern cropping regions of subtropical Australia. In Australia, *P. paradoxa* has evolved multiple herbicide resistance across two sites of action [“Fop” (aryloxyphenoxy propionates) and ALS group of herbicides]; therefore, there are reports of poor control of *P. paradoxa* with the use of these herbicides^6,8^.

Knowledge of the seed biology of weed*s* is essential for developing effective weed management strategies to control and delay the onset of herbicide resistance^9^. Seed germination behavior is an important factor for the establishment of weeds in cropping regions and understanding the weed seed ecology could play an important role in the decision-making process for weed control by formulating effective strategies^10^. Weed seed persistence in the soil is highly influenced by the germination and dormancy behaviors of the weeds under different environmental conditions^11^.

Despite the significant yield losses caused by *P. paradoxa* in Australian wheat production systems, knowledge regarding the seed germination ecology of *P. paradoxa* is limited. Weed seed persistence in a cropping region is always dependent on critical events of seed germination under different environmental conditions^2^. Environmental parameters, such as temperature, light, and water stress conditions, regulate seed germination^12–14^. As *P. paradoxa* is a significant weed in wheat crops, a better understanding of the germination and emergence behavior of *P. paradoxa* and wheat in response to environmental conditions (temperature, light, radiant heat, salinity, water stress, and burial depth) would be useful in developing effective management strategies for *P. paradoxa* in wheat.

Knowledge of optimum temperature conditions for weed seed germination could help in altering the sowing time of crops for reduced infestation of weeds^15^. Similarly, knowledge of the light requirement of seeds could help in managing weeds by using different tillage systems. It has been estimated that a 60% area of the Australian cropping region has sodic soils^16^. The accumulation of alkaline salts may increase the pH of many soils to as high as 9.0 ^17^. The germination response of *P. paradoxa* and wheat seeds in relation to these factors (e.g., salinity) may determine weed invasiveness, the extent of crop-weed competition, and the pattern of weed spread^18^. From an ecological point of view, therefore, it is important to evaluate the effect of these environmental conditions on the germination and emergence of *P. paradoxa* and wheat.

There may be differing germination responses for *P. paradoxa* and wheat in relation to temperature regimes^18^. For instance, germination in the closely related species *P. minor* was highest (96%) at a temperature regime of 25/15 ℃ (day/night) and nil at 35/25 ℃^19^. However, such information is very limited for *P. paradoxa* under Australian conditions.

It has been observed that dormancy in *P. paradoxa* seeds could be overcome by placing imbibed seeds in the dark at 16 ℃ for either 7 or 14 d, followed by exposure to red or white light for a 15-min period^2^. In another study on a closely related species, *P. minor*, it was observed that germination was reduced in dark environments compared with light conditions ^20^. Considering these differences in germination responses of *Phalaris* species under different light and temperature conditions, it is pertinent to evaluate the germination behavior of *P. paradoxa* and wheat in response to different temperature and light conditions. Therefore, it is possible that the temperature and light response of *P. paradoxa* may be similar or different from that of wheat. Seed burial depth has an important role in inducing depth-mediated dormancy, especially in small-sized weed seeds^21^. Therefore, a better understanding of the burial depth factor that could affect the germination of *P. paradoxa* under no-till systems is needed. Such understanding will help in developing effective cultural management practices for this problematic weed.

There are reports of poor control of *P. paradoxa* with ACCase inhibiting herbicides in Australia. A similar type of situation was also observed in central and southern Italy, and Mexico^22^. Therefore, there is a need to evaluate alternative pre-emergent (PRE) and post-emergent (POST) herbicides for the control of *P. paradoxa*. The efficacy of PRE herbicides may vary under different residue covers in paddocks due to the adsorption of herbicides^23^. The efficacy of POST herbicides may vary with the size of weed plants in the paddock. Therefore, it is pertinent to evaluate PRE and POST herbicides under these situations for better control of *P. paradoxa*.

The primary objective of this study was to evaluate the effects of temperature, light quantity, radiant heat, salt stress, water stress, and burial depth on seed germination between both *P. paradoxa* and wheat. A secondary objective was to evaluate the performance of different PRE and POST herbicides on *P. paradoxa* control.

## Material and methods

### Seed collection and multiplication

For experimental studies, seeds of *P. paradoxa* were initially collected from approximately 200 plants grown at Millmerran, Queensland, Australia (27.82625°S, 151.3513°E) in January 2018. For collecting seeds, permission was accorded from the consultants through phone calls and personal meetings. The spikes of *P. paradoxa* were collected at the maturity stage of plants. Seeds were then threshed from spikes by gentle rubbing on hands at the Weed Science Laboratory (27.5552°S, 152.343°E), Gatton, Australia. After cleaning, seeds were stored in paper bags at room temperature (25 °C). Seeds were multiplied in a screenhouse in 2019 by growing plants in pots using potting mix (Centenary Landscaping, Brisbane, Queensland, Australia). For the multiplication of seeds, plants of *P. paradoxa* were grown in May 2019, and seeds were collected in October 2019. Fresh seeds from the matured plants grown in 2019 were used for experimental studies. For studies on wheat, seeds of a commercial variety Spitfire were used. Each study was conducted two times. All local, national or international guidelines and legislation were adhered for the use of plants in this study.

### Effect of temperature

The study was conducted in a factorial, completely randomized design with three replications. The first factor was plant species (wheat and *P. paradoxa*) and the second factor was temperature regime [15/5, 20/10, 25/15, 30/20, and 35/25 °C (day/night temperatures 12 h/12 h)]. Treatment-wise, 25 seeds per treatment were sown in Petri dishes lined with double-layer WhatmanTM filter paper that was moistened with 5 mL of tap water. A micropipette (BOECO Germany) was used to add 5 mL water. After sowing, Petri dishes were put in sealable plastic bags. The bags were sealed and transferred to incubators (Labec Laboratory Pvt. LTD, Australia) at the respective temperature regime. Germination data were taken 21 d after sowing. Seeds were considered germinated when at least a 2 mm radical was observed.

### Effect of light intensity

The study was conducted in a factorial, completely randomized design with three replications. The first factor was two plant species: wheat (*Triticum aestivum* L.) and *P. paradoxa*; and the second factor was five light intensity levels (0, 30, 50, 70, and 100%). Seeds were sown in Petri dishes in a similar way as in the temperature experiment and were incubated at 20/10 °C (day/night 12 h/12 h temperature). For zero light intensity, Petri dishes were covered with double aluminum foils. For 30, 50, and 70% light intensity, Petri dishes were covered with meshes of different intensities. The light intensity was measured using a Quantum meter (MQ-200 Apogee Instruments, USA). Germination data were taken 21 d after sowing.

### Effect of radiant heat

To examine the effect of radiant heat on seed germination, 600 seeds of *P. paradoxa* were divided into six paper bags, each containing 100 seeds. The paper bags were labeled as per respective preheat treatments (25, 50, 100, 150, 200, and 250 °C). The hotter temperatures of the heat treatment were selected to simulate temperatures during the burning of crop straws in a field (Cook 1939). Seeds stored at room temperature were used for the 25 °C treatment and other treatments were conducted in an oven at specific temperatures for 5 min. After heat treatment, seeds were sown in Petri dishes in a similar way as in the temperature experiment and incubated at 20/10 °C. Germination data were taken 21 d after sowing.

### Effect of osmotic stress

The study was conducted in a factorial, completely randomized design with three replications. The first factor was plant species (wheat and *P. paradoxa*) and the second factor was water stress levels (0, −0.1, −0.2, −0.4, −0.8, and −1.6 MPa). Seeds were sown in Petri dishes in a similar way to the temperature experiment, but filter papers were moistened with 5 mL of different concentrations of osmotic potentials, which were prepared using polyethylene glycol (PEG 8000) (Sigma-Aldrich Co., St. Louis, MO). Seeds were incubated at 20/10 °C. Germination data were taken 21 d after sowing.

### Effect of salinity

The study was conducted in a factorial, completely randomized design with three replications. The first factor was plant species (wheat and *P. paradoxa*) and the second factor was salinity levels (0, 25, 50, 100, 150, and 200-mM sodium chloride, NaCl). Seeds were sown in Petri dishes in a similar way as in the temperature experiment, but filter papers were moistened with 5 ml of different concentrations of NaCl. Seeds were incubated at 20/10 °C. Germination data were taken 21 d after sowing.

### Effect of burial depth

For the burial depth experiment, field soil (after sieving through a 0.3 cm mesh) was used. The soil used was clay loam in texture, with organic matter comprising 2.7%, and a pH of 7.2. The experiment was conducted in pots having a diameter of 12 cm. Pots were filled with the sieved soil leaving the corresponding depth to the top of the pot as 0, 0.5, 1, 2, 4, and 8 cm. Forty seeds of *P. paradoxa* were sown in each pot and covered with the same soil to achieve the desired depth. The experiment was conducted in a completely randomized design and each treatment was replicated thrice. The pots were regularly irrigated using a sprinkler system to avoid any moisture stress. Seedling emergence data were collected at 28 d after sowing. Seedlings were considered emerged when the coleoptiles were seen above the soil surface.

### Evaluation of PRE-herbicides

The study was conducted in a factorial, completely randomized design with three replications. The first factor was plant species (wheat and *P. paradoxa*) and the second factor was herbicide treatment (pendimethalin 594 g ai ha^−1^, triallate 800 g ai ha^−1^, trifluralin 384 g ai ha^−1^, atrazine 585 g ai ha^−1^, pyraxosulfone 100 g ai ha^−1^, s-metolachlor 240 g ai ha^−1^, isoxaflutole 75 g ai ha^−1^, dimethenamid-P 720 g ai ha^−1^, imazethapyr 70 g ai ha^−1^, prosulfocarb + s-metolachlor 2300 g ai ha^−1^, bixlozone 500 g ai ha^−1^, and cinmethylin 375 g ai ha^−1^). The study was conducted in 12 cm diameter pots filled with potting mix. Twenty seeds were sown in each pot. After sowing, seeds were covered with a thin layer (0.5 cm) of potting mix. Spraying was done using a research track sprayer equipped with Teejet XR 110,015 flat fan nozzles calibrated to an output spray volume of 108 L ha^-1^. Plant survival was recorded 28 d after sowing.

### Efficacy of PRE-herbicides in relation to crop residue cover

The study was conducted in a factorial, completely randomized design with three replications. The first factor was crop residue level (0, 2, and 6 t ha^−1^) and the second factor was PRE herbicide (pyraxosulfone 100 g ai ha^−1^, cinmethylin 375 g ai ha^−1^, triallate 800 g ai ha^−1^, pendimethalin 594 g ai ha^−1^, isoxaflutole 75 g ai ha^−1^, imazethapyr 70 g ai ha^−1^). Pots (12 cm diameter) were filled with potting mix and 20 seeds were sown in each pot. After sowing, seeds were covered with chopped (about 2 cm in length) wheat residue according to treatment. The residue was used after oven drying the wheat straw in an oven at 70 C for 72 h. Herbicide spray was done using a research track sprayer equipped with Teejet XR 110,015 flat fan nozzles calibrated to an output spray volume of 108 L ha^−1^. Data on plant survival was recorded 28 d after sowing.

### Efficacy of POST herbicides in relation to plant size

The study was conducted in a factorial, completely randomized design with three replications. The first factor was the size of the plant (4-leaf and 10-leaf stages) and the second factor was herbicide treatment (clodinafop 20 g ai ha^−1^, propaquizafop 30 g ai ha^−1^, clethodim 120 g ai ha^−1^, butroxydim 45 g ai ha^−1^, pinoxaden 20 g ai ha^−1^, haloxyfop 78 g ai ha^−1^, imazamox + imazapyr 36 g ai ha^−1^, glyphosate 741 g ae ha^−1^, glufosinate 750 g ai ha^−1^ and paraquat 600 g ai ha^−1^). For this study, pots (12 cm diameter) were filled with potting mix and 10 seeds were sown in each pot. After emergence, only 6 plants per pot were maintained. Plants were watered regularly to avoid any water stress. For larger plants (10-leaf stage), sowing was done two weeks earlier than the 4-leaf stage plants, so that herbicide spray could be done on the same day. Herbicide spray was done using a research track sprayer equipped withTeejet XR 110,015 flat fan nozzles calibrated to an output spray volume of 108 L ha^−1^. Data on plant survival was recorded 28 days after sowing.

### Statistical analyses

Each experiment was repeated once over time. Each dataset represents the average of the two runs, as there was no time by treatment interaction as determined by analysis of variance (ANOVA). Except for the PRE herbicide x residue experiment, the homogeneity of variance was not improved by transformation, and residuals were normally distributed; thus, ANOVA was performed on nontransformed germination percentage values (using GENSTAT 16th Edition; VSN International, Hemel Hempstead, UK). Survival data in the PRE herbicide x residue experiment were subjected to square-root transformation (√x + 0.5) before analysis. ANOVA was used to identify any significant treatment and interaction effects (P ≤ 0.05). Where treatments were significant (P ≤ 0.05), means were separated using Fisher’s protected LSD test.

## Results and discussion

### Effects of light intensity and temperature

The germination of *P. paradoxa* (91 to 95%) and wheat (93 to 97%) was not affected by light intensity (data not shown). Our results conform to previous studies which revealed that light intensity had little role in influencing *P. paradoxa* germination^24^.

**Figure 1:**
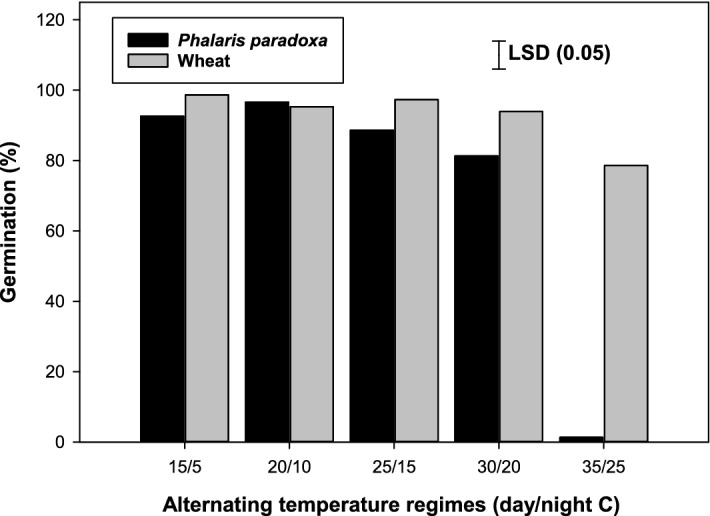
Effect of alternating day/night temperatures (15/5 to 35/25 °C) on germination of *Phalaris paradoxa* and wheat seeds (incubated for 21 d) under light/dark (12-h photoperiod). LSD: Least significant difference at the 5% level of significance.

The germination of wheat and *P. paradoxa* was influenced by temperature regimes (Fig. 1). At temperature regimes of 15/5 °C and 20/10 °C, germination of wheat and *P. paradoxa* did not vary. Seed germination in wheat remained similar at temperatures ranging between 15/5 °C to 30/20 °C. However, in *P. paradoxa*, germination was reduced at higher temperature regimes (35/25 C) compared with lower temperature regimes (15/5 °C to 25/15 °C). At the highest temperature regime (35/25 °C), the germination of wheat was 79%, while, at this temperature regime, the germination of *P. paradoxa* was only 1%. This suggests that wheat can germinate at high-temperature ranges, while, germination of *P. paradoxa* may be reduced at high temperatures (35/25 °C). These results implied that at the time of planting wheat in Australia if the air temperature is low, the chances of emergence of *P. paradoxa* are very high. This suggests that efforts should be made towards early control of *P. paradoxa* in wheat if the air temperature in the winter season falls early. These results also suggest that early planting of wheat could reduce the emergence of *P. paradoxa* as the prevailing temperature conditions are relatively high in early planting (e.g., end of April). In the Indo-Gangetic Plains, better control of *P. minor* was observed in the early planting of wheat (high-temperature conditions) due to less emergence of *P. minor*^25^.

Previous studies have also revealed that germination of *P. paradoxa* was highest at 10 °C and then failed to germinate at 30 °C ^24,26^, however, these studies were conducted at constant temperatures and the germination response of *P. paradoxa* was not studied in comparison with wheat in those studies.

### Effect of radiant heat

**Figure 2:**
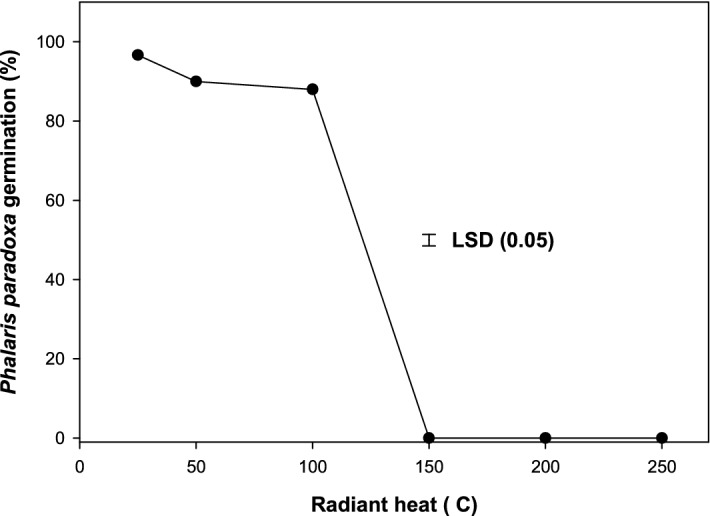
Effect of high-temperature pretreatment for 5 min (℃) on germination of *Phalaris paradoxa* seeds. LSD: Least significant difference at the 5% level of significance.

The germination of *P. paradoxa* seeds that were stored at room temperature (25 °C) was 97%, which reduced to 88% after exposure to the 100 °C pretreatment for 5 min and became nil at 150 °C (Fig. 2). About 88% of *P. paradoxa* at 100 °C suggests that it can tolerate heat stress for short periods.

Germination was nil at 150 °C and above, suggesting that burning could help in managing *P. paradoxa*, particularly in a no-till field where seeds are on the soil surface or at shallow depths. Exposure of seeds to fire could inhibit germination by desiccating the seed coat or by damaging the embryo^27–29^.

Burning of residue in the fields could kill weed seeds and other pests in the topsoil layer^30^. Windrow burning proved to be an effective tool for killing weed seeds in paddocks^31^. However, the crop residue burning may cause environmental destruction by killing microbes and polluting the air. Also, it reduces the amount of soil organic matter due to the high heat, causing soil degradation. Therefore, these aspects should also be considered while formulating weed management strategies through crop residue burning. Burning may also release the dormancy of other weed seeds present in the subsoil and thus may increase infestation; therefore, this technique should be used cautiously^32,33^.

### Effect of osmotic stress

**Figure 3:**
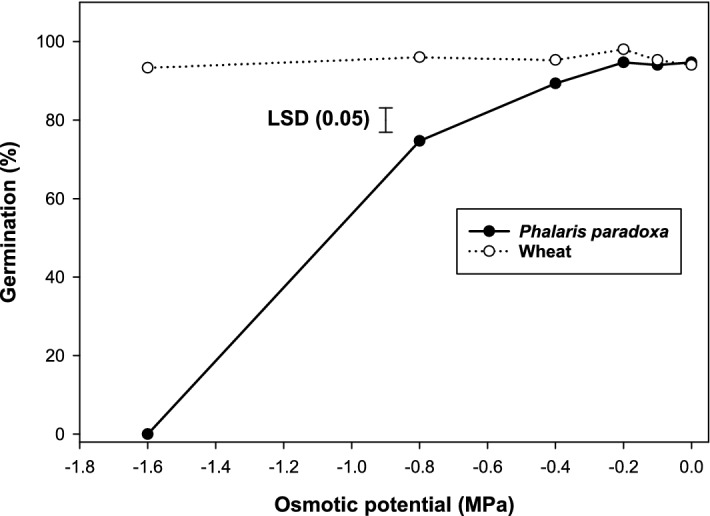
Effect of osmotic potential on germination of *Phalaris paradoxa* and wheat seeds at alternating day/night temperatures of 20/10 °C under 12 h photoperiod. Seeds were incubated for 21 d. LSD: Least significant difference at the 5% level of significance.

Germination of *P. paradoxa* was highest (95%) in the control treatment and germination reduced to 75% at an osmotic potential of −0.8 MPa, and became nil at −1.6 MPa (Fig. 3). However, in wheat, germination did not reduce with an increase in water potential and it was 94% in the control treatment.

At a very high concentration of PEG, the metabolic activity of *P. paradoxa* might be reduced due to water stress. Seed germination is affected when seeds are not able to get critical moisture threshold levels for imbibitions^34,35^. These results indicate that high water stress may inhibit the seed germination of *P. paradoxa*. However, under no water stress or mild water stress conditions, *P. paradoxa* may infest the wheat crop.

Contrary to these results, previous studies reported that germination of *P. paradoxa* was reduced by 90% at an osmotic potential of −0.25 MPa^25^. Good germination of wheat at high osmotic potential indicates that the wheat variety used in this study may have water stress tolerance traits for germination. It was observed that wheat could germinate well (75%) at a high-water stress level (−1.6 MPa)^36^. This suggests that it is possible to menace *P. paradoxa* by growing stress-tolerant varieties of wheat and manipulating irrigation. In a previous study, less infestation of *P. paradoxa* was observed in drip-irrigated wheat crops due to optimal soil moisture conditions for the crop^37^.

### Effect of salt stress

**Figure 4:**
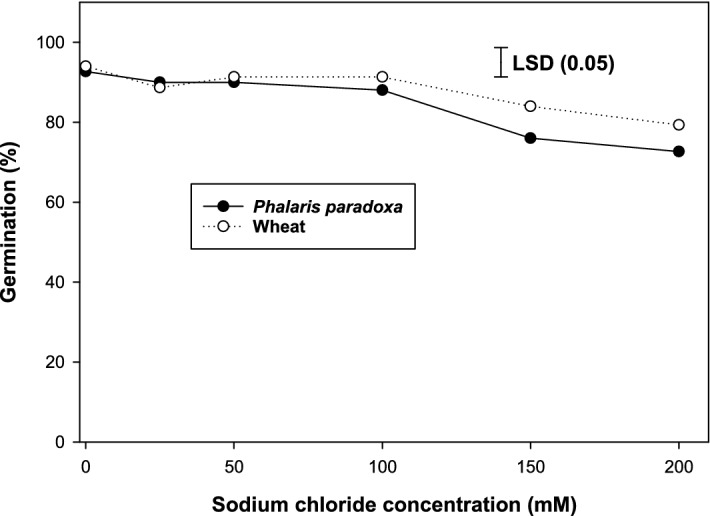
Effect of sodium chloride concentration on germination of *Phalaris paradoxa* and wheat seeds at alternating day/night temperatures of 20/10 °C under 12 h photoperiod. Seeds were incubated for 21 d. LSD: Least significant difference at the 5% level of significance.

Germination of *P. paradoxa* was highest (93%) in the control treatment, and at a NaCl of 150 mM, germination was reduced to 76% (Fig. 4). Similarly, in wheat, germination was highest (94%) in the control treatment and at a salt concentration of 150 and 200 mM, germination was reduced to 84 and 79%, respectively. These results suggest that at a high salt concentration, *P. paradoxa* may infest the wheat crop owing to its ability to germinate under high salt concentrations.

Contrary to this, in Iran, it was observed that germination of *P. paradoxa* was reduced by 70% at a NaCl of 160 mM^24^. Most of the Australian soils are saline; therefore, it is quite possible that *P. paradoxa* in Australia might have developed traits for salt tolerance^38^. The variable response of populations of *P. paradoxa* to salt concentrations in Iran and Australia might be due to genetic differences between the *P. paradoxa* populations^38^. These observations suggest that *P. paradoxa* could invade the agroecosystem under the saline conditions of Australia.

### Effect of seed burial depth on emergence

**Figure 5:**
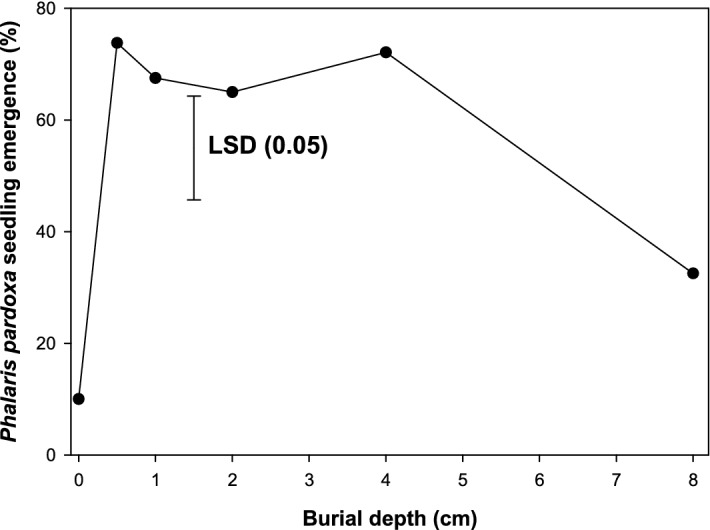
Effect of seed burial depth on seedling emergence of *Phalaris paradoxa.* LSD: Least significant difference at the 5% level of significance.

Germination of *P. paradoxa* was very low (10%) on the soil surface, and seedling emergence was highest (74%) at a soil burial depth of 0.5 cm (Fig. 5). Seedling emergence was similar when seeds were buried in the soil at a depth ranging from 0.5 to 4 cm. Seedling emergence was 32% at a burial depth of 8 cm.

The results from this experiment suggest that a no-till production system may inhibit the germination of *P. paradoxa*. This study also suggests that deep tillage (> 4 cm) could reduce the emergence of *P. paradoxa* to some extent; therefore, inversion tillage could be a weed management strategy if the seedbank is in the shallow layer of the soil. It has been reported that the emergence of small-seeded weeds is reduced from deeper burial depths, as the soil-gas exchange is limited ^21^. However, it is important to know the seed longevity of this weed in different soil and environmental conditions when considering tillage operations^39^.

Likewise, previous studies also reported that seed germination of *P. paradoxa* was lowest on the soil surface and no seedlings emerged from a soil depth of 10-cm^2,40^. Contrary to this in Iran, germination of *P. paradoxa* was found to be > 65% on the soil surface ^24^.

### Evaluation of PRE-herbicides

Results revealed that cinmethylin, pyroxasulfone, and trifluralin provided 100% control of *P. paradoxa*. Atrazine, bixlozone, imazethapyr, isoxaflutole, prosulfocarb + s-metolachlor, and s-metolachlor were not found to be effective against *P. paradoxa* (Table 1). Pendimethalin and triallate controlled *P. paradoxa* by 80 and 42%, respectively, compared with the nontreated control.

**Table 1:** Effect of PRE herbicides on the survival of *Phalaris paradoxa* and wheat seedlings (28 d after spray).

Herbicide treatments (g a.i. ha^-1^)	Plant survival (%)	
	*P. paradoxa*	Wheat
Control	89.2d	95.8de
Atrazine 585	85d	88.3d
Bixlozone 500	89.1d	92.5d
Cinmethylin 375	0a	81.7d
Dimethenamid-P 720	8.3a	56.7c
Imazethapyr 70	84.2d	95de
Isoxaflutole 75	88.3d	95de
Pendimethalin 594	17.5ab	90d
Prosulfocarb + s-metolachlor 2300	85.8d	80.8d
Pyraxosulfone100	0a	87.5d
S-metolachlor 240	80.8d	93.3d
Triallate 800	51.7c	86.7d
Trifluralin 384	0a	93.3d
LSD (0.05)	12.7	

The means sharing similar letter(s) are statistically similar.

In wheat, all tested herbicides performed similarly for plant survival except dimethenamid-P and prosulfocarb + s-metolachlor, which caused wheat mortality by 41 and 16%, respectively, compared with the nontreated control. These results suggest that pyroxasulfone, pendimethalin, and trifluralin can be successfully used for the management of *P. paradoxa* in wheat. Alternative use of these herbicides in wheat crops could provide sustainable weed control of *P. paradoxa*. In previous studies conducted in Australia, herbicides namely cinmethylin, pyroxasulfone, and trifluralin were found safe for wheat and provided excellent grass weed control^41^.

### Efficacy of PRE-herbicides in relation to crop residue cover

Cinmethylin, pendimethalin, and pyroxasulfone were proven to be very effective against *P. paradoxa* under no residue cover conditions (Table 2). However, at the residue cover of 6 t ha^-1^ (high output systems), the efficacy of these herbicides decreased and these three herbicides failed to provide effective control of *P. paradoxa.* At the residue cover of 2 t ha^-1^ (low output systems), the efficacy of pyroxasulfone in controlling *P. paradoxa* was not affected; however, cinmethylin and pendimethalin at the residue load of 2 t ha^-1^ did not control *P. paradoxa*. These results suggest that in a residue-retained, no-till system, pyroxasulfone could provide better control of *P. paradoxa* compared with cinmethylin and pendimethalin.

**Table 2:** The interaction of PRE herbicides and wheat residue amount on the survival of *Phalaris paradoxa* seedlings at 28 d after spray.

Herbicide treatments (g ai ha^-1^)	*Phalaris paradoxa* survival (%)		
	Wheat residue amount (t ha^-1^)		
	0	2	6
Control	4.28 (23.3)ab	7.31 (55.8)bc	9.08 (82.5)c
Cinmethylin 375	2.94 (9.2)a	6.65 (46.7)b	7.81(61.7)bc
Imazethapyr 70	5.60 (40.8)b	7.51 (57.5)bc	8.74 (76.7)bc
Isoxaflutole 75	5.62 (38.3)b	6.81 (50.8)b	8.81 (78.3)bc
Pendimethalin 594	2.50 (10)a	6.50 (44.2)b	8.51 (72.5)bc
Pyraxosulfone 100	1.27 (3.3)a	1.89 (7.5)a	6.11 (39.2)b
Triallate 800	3.53 (14.2)ab	7.29 (55.8)b	8.44 (71.7)bc
LSD (0.05)	1.70		

The means sharing similar letter(s) are statistically similar.

Weed survival data were subjected to square-root transformation before analysis. Original values are shown in parentheses.

The crop residue binds some herbicides, which results in a reduced dose to target weeds and provides poor weed control^42^. A crop residue cover of 1 t ha^-1^ may prevent 50% of the herbicide from reaching the target weed seeds in the soil and thus provide poor weed control^43^.

### Efficacy of POST herbicides in relation to plant size

When plants were sprayed at the 4-leaf stage, the herbicides clodinafop and propaquizafop were not effective against *P. paradoxa* compared with the other tested herbicides (Table 3). The efficacy of clethodim, glyphosate, haloxyfop, and paraquat in controlling *P. paradoxa* was not decreased even when plants were sprayed at the 10-leaf stage. In previous studies, poor control of *P. paradoxa* was observed with ACCase-inhibiting herbicides^44,45^. These results also suggest that under noncropped or fallow situations, early and late cohorts of *P. paradoxa* can be controlled successfully by delaying applications of clethodim, paraquat, haloxyfop, and glyphosate.

**Table 3:** The interaction effect of plant size (large plants-10 leaves and small plants-4 leaves) and herbicide treatments on the survival of *Phalaris paradoxa* seedlings at 28 d after spray.

Treatment (g a.i./a.e. ha^-1^)	Plant survival (%)	
	Large plants	Small plants
Control	100c	100c
Butroxydim 45	72.8bc	0a
Clethodim 120	0a	0a
Clodinafop 20.4	100c	56.1b
Glufosinate 750	91.1bc	50b
Glyphosate 741	0a	0a
Haloxyfop 78	6.1a	0a
Imazamox + imazapyr 36	100c	0a
Paraquat 600	0a	0a
Pinoxaden 20	83.9c	0a
Propaquizafop 30	86.7c	46.7b
LSD (0.05)	22.0	

The means sharing letter(s) are statistically similar.

Germination of *P. paradoxa* at 25/15 °C (day/night) was lower compared with 20/10 °C. This suggests that early sowing of wheat (relatively high-temperature conditions) could reduce the emergence of *P. paradoxa* in fields. *Phalaris paradoxa* did not germinate after exposure to radiant heat of 150 °C (for 5 min), which suggests that burning may be a useful tool for managing *P. paradoxa*, particularly when seeds are on the soil surface or at the shallow surface. A high level of tolerance of *P. paradoxa* to water and salt stress was observed. These observations suggest that this weed can dominate under saline and water stress conditions in Australia. Low germination of *P. paradoxa* was observed on the soil surface, suggesting that a no-till system could provide better control of *P. paradoxa*. PRE herbicides cinmethylin, pyroxasulfone, pendimethalin, and trifluralin were effective for control of *P. paradoxa* in wheat; however, under a conservation tillage system, pyroxasulfone provided better control of *P. paradoxa* compared with other herbicides. Haloxyfop and clethodim were the most effective herbicides among the ACCase-inhibiting herbicides. Under noncropped or fallow land situations, larger plants of *P. paradoxa* can be successfully controlled with the application of clethodim, glyphosate, and paraquat.

## Supplementary Information

Supplementary Information.

## Data Availability

All relevant data are within the paper and its Supporting Information files.
